# Targeted Small-Molecule Identification Using Heartcutting Liquid Chromatography–Infrared Ion Spectroscopy

**DOI:** 10.1021/acs.analchem.2c04904

**Published:** 2023-02-03

**Authors:** Rianne E. van Outersterp, Jitse Oosterhout, Christoph R. Gebhardt, Giel Berden, Udo F. H. Engelke, Ron A. Wevers, Filip Cuyckens, Jos Oomens, Jonathan Martens

**Affiliations:** † Radboud University, Institute for Molecules and Materials, FELIX Laboratory, Toernooiveld 7, 6525 ED Nijmegen, The Netherlands; ‡ Bruker Daltonik GmbH & Co. KG, Fahrenheitstrasse 4, D-28359 Bremen, Germany; § Department of Laboratory Medicine, Translational Metabolic Laboratory, Radboud University Medical Center, 6525 GA Nijmegen, The Netherlands; ∥ Drug Metabolism & Pharmacokinetics, Janssen R&D, Beerse 2340, Belgium; ⊥ van’t Hoff Institute for Molecular Sciences, University of Amsterdam, 1098XH Amsterdam, The Netherlands

## Abstract

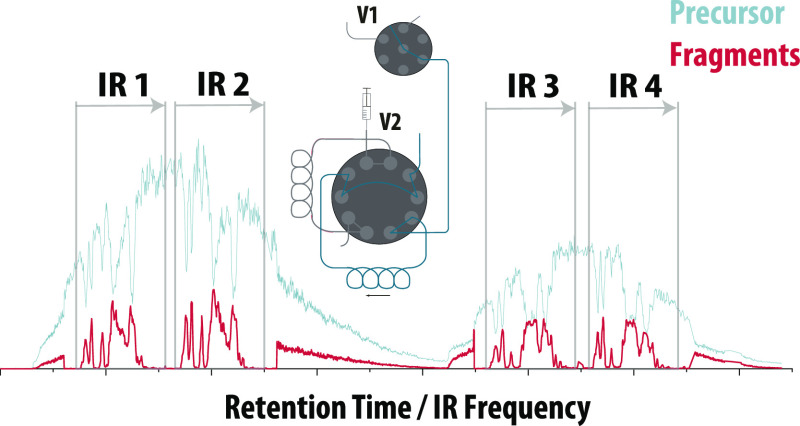

Infrared ion spectroscopy (IRIS) can be used to identify molecular structures detected in mass spectrometry (MS) experiments and has potential applications in a wide range of analytical fields. However, MS-based approaches are often combined with orthogonal separation techniques, in many cases liquid chromatography (LC). The direct coupling of LC and IRIS is challenging due to the mismatching timescales of the two technologies: an IRIS experiment typically takes several minutes, whereas an LC fraction typically elutes in several seconds. To resolve this discrepancy, we present a heartcutting LC-IRIS approach using a setup consisting of two switching valves and two sample loops as an alternative to direct online LC-IRIS coupling. We show that this automated setup enables us to record multiple IR spectra for two LC-features from a single injection without degrading the LC-separation performance. We demonstrate the setup for application in drug metabolism research by recording six m/z-selective IR spectra for two drug metabolites from a single 2 μL sample of cell incubation extract. Additionally, we measure the IR spectra of two closely eluting diastereomeric biomarkers for the inborn error of metabolism pyridoxine-dependent epilepsy (PDE-ALDH7A1), which shows that the heartcutting LC-IRIS setup has good sensitivity (requiring ∼μL injections of ∼μM samples) and that the separation between closely eluting isomers is maintained. We envision applications in a range of research fields, where the identification of molecular structures detected by LC–MS is required.

## Introduction

Mass spectrometry (MS) is among the most popular analytical methods employed in a range of scientific fields, which can be attributed to its ultrahigh sensitivity and selectivity as compared to alternative techniques, most notably nuclear magnetic resonance spectroscopy. This allows for the detection of low-abundance analytes from complex mixtures, such as body fluids, containing thousands of molecular species. However, the amount of molecular structure information that can be extracted from an MS experiment is often limited. Each detected *m*/*z*-feature can potentially correspond to a number of structural isomers and determining which of these is present in a given sample is often nontrivial.^1−3^ To address this, a range of orthogonal technologies hyphenated to MS have been developed, such as fragmentation technologies,^4^ ion mobility,^5^ and (liquid and gas) chromatography.^6,7^

Infrared ion spectroscopy (IRIS)^8−11^ is a relatively novel approach integrating MS with infrared spectroscopy, so that an infrared spectrum, and hence direct information on chemical bonding, can be obtained for an isolated *m*/*z*-feature. Recent studies have detailed its potential for molecular structure elucidation in a variety of research fields including forensics,^12−14^ environmental science,^15−17^ drug development,^18−20^ and clinical research.^18,21,22^ IRIS is an action spectroscopy technique; IR spectra are generated by recording the photodissociation of ions after irradiation at a series of IR laser frequencies. Plotting the extent of fragmentation as a function of the IR frequency provides the IR spectrum. IRIS can therefore be incorporated in an MS-based analytical workflow analogous to other fragmentation MS/MS methods. However, to record an entire IR spectrum, individual MS/MS spectra need to be recorded at each IR frequency of the laser (e.g., 500–2000 cm^–1^ in 3–5 cm^–1^ steps), which typically involves the acquisition of 300–500 MS/MS spectra and all together requires >10 min. Previous work has shown that for experiments distinguishing between a few targeted “known unknowns”, spectral libraries can be constructed that allow the distinction of compounds based on only a few isomer-specific wavelengths making the experiment much faster. However, more extensive IR spectra covering a broader frequency range are typically required for the identification of unknowns.

To avoid ionization suppression effects and to separate isomers, MS is often combined with an analytical separation technique in the analysis of complex biochemical samples. Liquid chromatography (LC) is the most widely used separation method, mainly due to its robustness and versatility in separating a wide variety of (nonvolatile) chemical structures.^6,23,24^ However, the direct coupling of LC and IRIS is not trivial, due to the mismatching time-scales of the experiments; peak widths in state-of-the-art ultrahigh pressure LC (UHPLC) separations are usually not longer than several seconds, much shorter than the ∼10 min required for the acquisition of an IRIS spectrum. To address this challenge, several approaches to LC-IRIS coupling have been proposed. Offline coupling involving fraction collection^20,25^ provides the highest flexibility for both the LC- and IRIS-experiments but has drawbacks in terms of potential sample loss, (oxidative) degradation and/or contamination, often giving an overall reduction in sensitivity related to collection and transfer of fractions to a syringe for direct infusion measurements. In contrast, online coupling gives the best potential for high-throughput methods but puts severe limitations on either the resolution of the LC separation or the number of MS/MS spectra that can be acquired and thus the IR frequency range that can be covered.^18^ As a third approach, a hybrid solution based on semionline coupling via stop flow LC-IRIS has been demonstrated, although this also necessitates sacrifices in separation performance and sensitivity.^26^

Here, we demonstrate heartcutting LC-IRIS (analogous to heartcutting 2D-LC approaches) as an alternative approach. This involves an interface between the LC and MS systems consisting of two switching valves and two sample loops, comparable to the interface commonly used between the two LC-dimensions in 2D-LC systems.^27−30^ With this setup, two fractions of eluent (containing separated *m*/*z* features of interest) from a single LC injection can be stored in sample loops and separately infused into the MS-instrument at reduced flow rates compared to those used for the LC separation. We demonstrate that this can provide ∼20 min of stable ion signal, which allows for the acquisition of multiple IR spectra for each analyte.

## Experimental Section

### Sample Preparation

Reference standards of phenylalanine and caffeine were dissolved in LC–MS grade H_2_O (∼1 μM) and directly used for LC-IRIS analysis. Midazolam was incubated at 10 μM for 120 min in human hepatocytes. The final hepatocyte density was 10^6^ cells/ml. A 40 μL incubation volume was quenched with two volumes of ACN/HCOOH 90:10 [v/v], and the sample was centrifuged for 10 min at 4000 rpm. The supernatant was used for analysis. A plasma sample from a patient with the inborn error of metabolism pyridoxine-dependent epilepsy (PDE-ALDHA7A1) was prepared as described before (see the SI for details).^31^

### Liquid Chromatography

LC separations were performed with a Bruker Elute UHPLC system consisting of a binary pump, a cooled autosampler, and a column oven. The outlet of the column was connected to the heartcutting LC-IRIS interface (see below). Separations were performed using a previously described standard method with minor modifications (see the SI for details).^31^

### Heartcutting Liquid Chromatography–Infrared Ion Spectroscopy Interface

**Figure 1:**
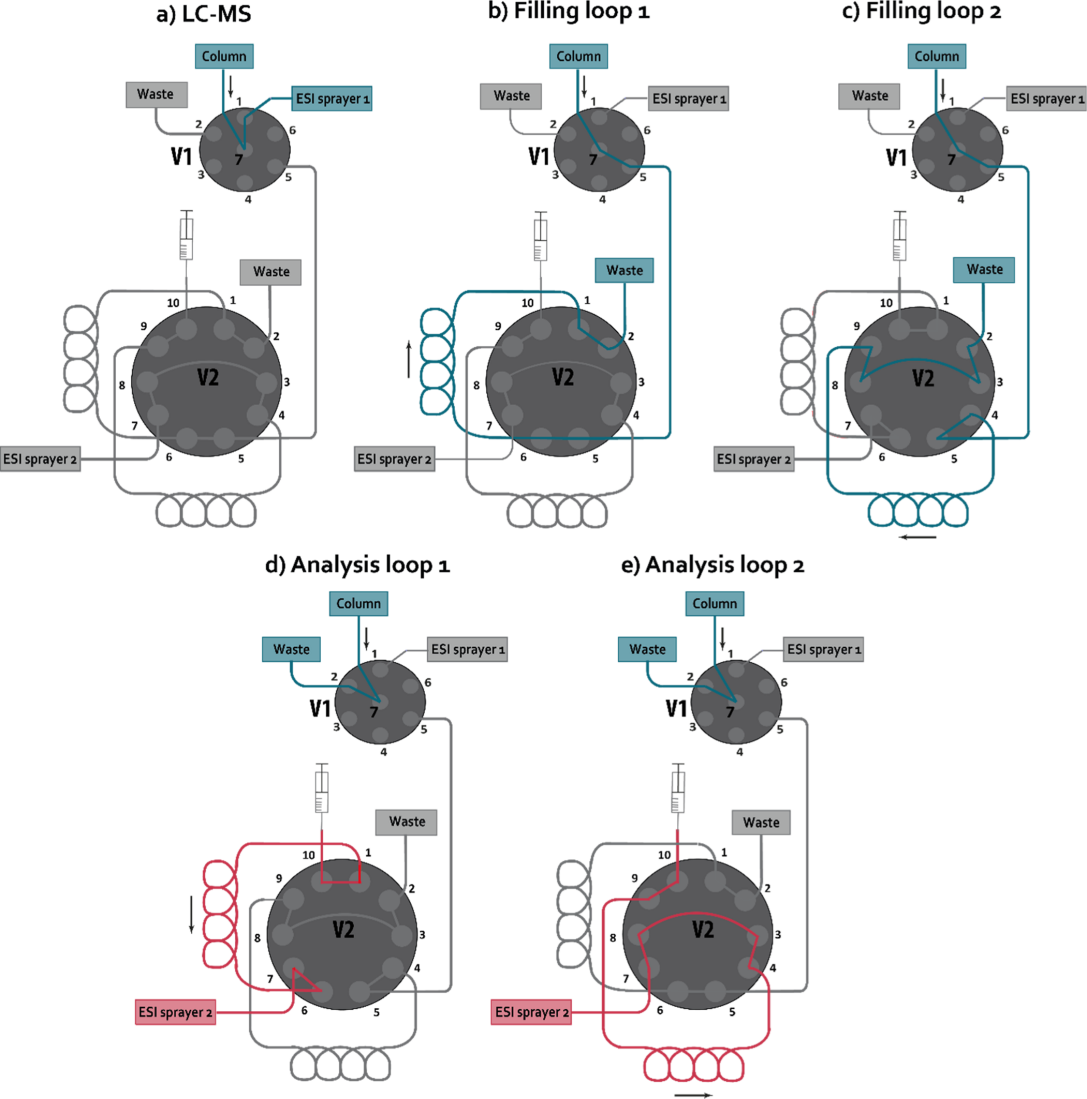
Schematic representation of the heartcutting LC-IRIS interface consisting of a six-position seven-port selector valve (V1) and a two-position 10-port switching valve (V2). The valves can be positioned to (a) perform regular LC–MS analysis, (b) fill the first sample loop with eluent, (c) fill the second sample loop with eluent, (d) infuse the contents of the first sample loop to the MS instrument, or (e) infuse the contents of the second sample loop to the MS instrument.

A schematic representation of the heartcutting LC-IRIS interface is shown in Figure 1. It consists of a six-position seven-port selector valve (PD7970 TitanHP pod, Idex) and a two-position 10-port switching valve (PD9960 TitanHP pod, Idex). Both valves and drivers (MHP0267–500-1 TitanHP actuator, Idex) were installed in a home-built switching box (see Figure S1a) which is under hardware control of the PC used to operate the MS via a USB connection. Experimental synchronization is done via an in-house developed LabView program sending down commands to the switching box and ion trap via the low-level Atlas development interface. The program contains a timed command queue that controls all events, which starts when the ion trap control program (trapControl, Bruker) switches to acquisition mode (i.e., at the start of an LC–MS experiment). Required user input is a timetable of events (i.e., switching of a specific valve) that take place during an experimental run.

The column outlet is connected to position 7 of the selector valve (V1, see Figure 1), sending the eluent to the ESI source of the ion trap (panel a), the second valve (V2, panel b and c) or waste (panel d and e). First, V1 is in the first position (7–1, panel a), allowing regular LC–MS analysis. When a fraction containing an ion of interest elutes from the column, V1 is switched (7–5, panel b) and the eluent flows to V2, which is equipped with two sample loops. The eluent fraction is stored in one of the sample loops and, subsequently, V1 is switched back to continue LC–MS analysis. When a second peak of interest elutes, both V1 and V2 are switched (panel c), filling the second sample loop. Subsequently, V1 can be switched back to monitor the final part of the LC run. This step may be skipped to save time. For IRIS analysis, V1 is switched (7–2, panel d) to send the eluent to waste. The column can be equilibrated to prepare for the next injection. The syringe, installed on the V2–10 position, is turned on to deliver a constant flow rate that is significantly lower than the LC-flow rate. Depending on the position of V2, the fraction stored in either one of the two loops (panel d or panel e) is slowly infused to the ESI source, generating an ion signal for IRIS analysis. Note that the loops are filled and emptied in opposite direction (“backflush” mode) to minimize band spreading. The sample loops are cleaned after IRIS analysis by switching V1 and V2 such that the mobile phase flows through each of the loops for a few minutes.

The electrospray ionization (ESI) source installed on the ion trap receives flow from two different parts of the setup ( V1–1 and V2–7, see Figure 1). To enable this, we modified the standard Apollo ESI source of the amaZon speed ion trap MS. Details of the modifications are given in the SI.

## Results and Discussion

### Evaluation of the Heartcutting LC-IRIS Setup

**Figure 2:**
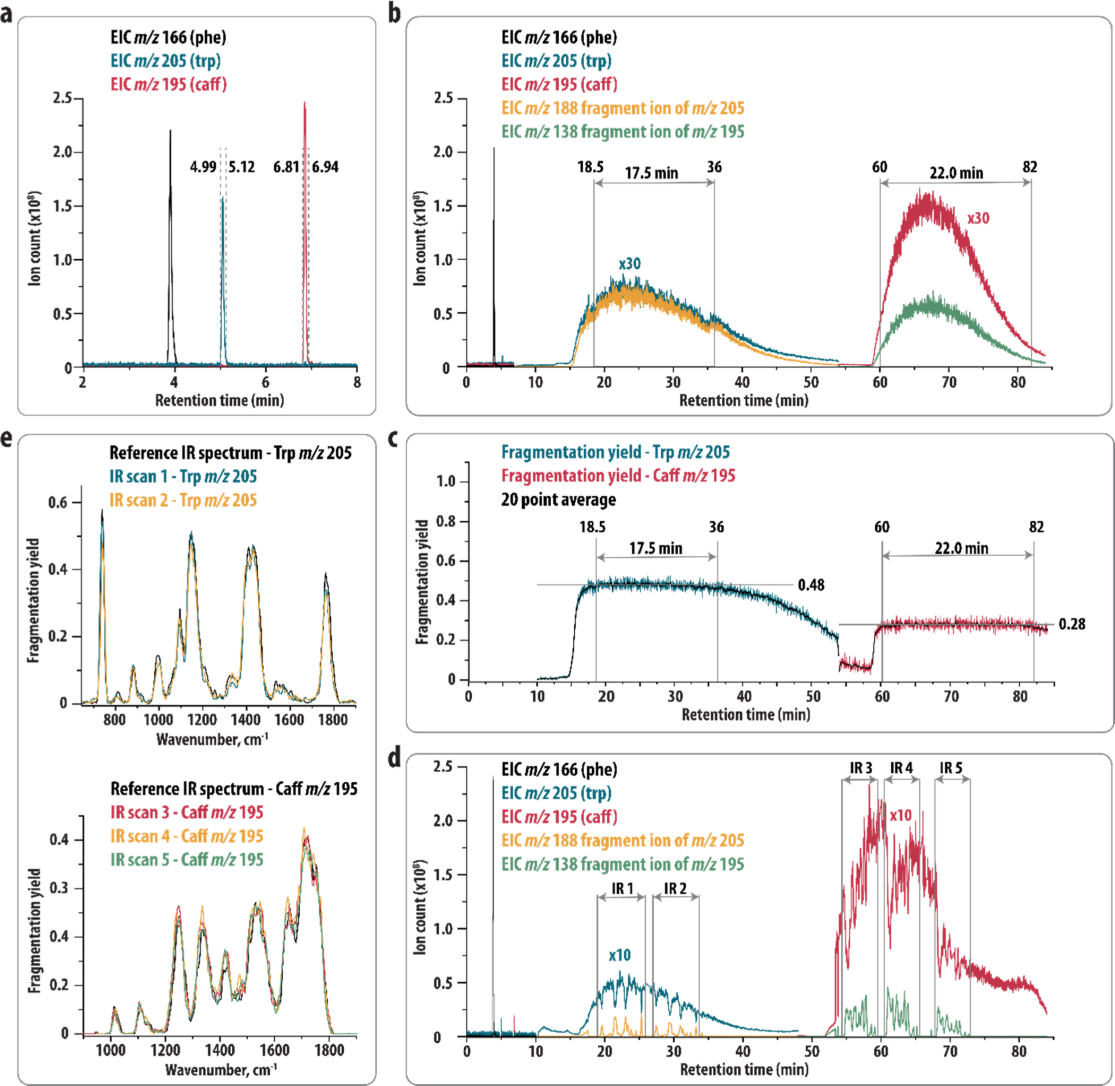
(a) EICs resulting from the LC–MS analysis of a mixture of Phe (*m*/*z* 166), Trp (*m*/*z* 205), and Caff (*m*/*z* 195). Switching times of the valves leading to storage of the Trp and Caff fraction in sample loops 1 and 2, respectively, are indicated (dashed lines). (b) EICs resulting from the analysis of the mixture using the heartcutting LC-IRIS set-up and CID with a constant amplitude. EICs during the loop analysis (>6.94 min) are scaled by 30 for better visualization. (c) Fragmentation yield of Trp and Caff observed during the analysis shown in (b). The region with a stable fragmentation yield is indicated. (d) EICs resulting from the analysis of the mixture using the heartcutting LC-IRIS setup and IRIS. The start and end of each IR scan are indicated. EICs during the analysis of the loops (>10 min) are scaled by 10 for better visualization. (e) IR spectra resulting from the IRIS analysis shown in (d) compared to reference IR spectra obtained in a direct infusion experiment.

We prepared a mixture (∼1 μM in H_2_O) of tryptophan, phenylalanine, and caffeine to evaluate the performance of the heartcutting LC-IRIS setup. All compounds were primarily detected as protonated structures ([M + H]^+^) in positive ESI mode (+ESI). The heartcutting LC-IRIS setup allows to record IR spectra, but also to record LC–MS chromatograms (with gaps corresponding to the heartcuts) in the same experiment. These can be used for quality-control purposes (i.e., to detect bad injections or retention time drifts). We used the signal of protonated phenylalanine (*m*/*z* 166) to demonstrate this. The peaks containing protonated tryptophan (*m*/*z* 205) and protonated caffeine (*m*/*z* 195) were stored in the sample loops for MS/MS analysis. In MS/MS experiments (both CID and IRMPD), protonated tryptophan produces a main fragment at *m*/*z* 188 and caffeine at *m*/*z* 138. Reference IRIS spectra of both compounds recorded using direct-infusion ESI are shown in Figure 2e. The mixture was separated using reversed-phase LC–MS (see the Experimental Section), and extracted ion chromatograms (EICs) of *m*/*z* 166 (phenylalanine), *m*/*z* 205 (tryptophan), and *m*/*z* 195 (caffeine) are shown in Figure 2a with retention times of ∼3.9, ∼5.1, and ∼ 6.9 min, respectively (the peak widths are ∼0.13 min). To set up an LC-IRIS experiment, the sample loop size and valve switching times need to be determined. Note that the linear velocity of solvent flowing through a small tube is approximately twice as high at the center of the tube as the average linear velocity (laminar flow^28,32^). A sample loop that exactly fits the LC peak (loop size = LC flow rate × peak width) therefore leads to sample losses. On the other hand, we found that when a sample loop is too large, i.e., if parts of the loop are filled with background mobile phase eluent, interference with isobaric background ions can occur. Therefore, we selected sample loops that are 30 percent larger than the theoretical size of the analyte fraction (following best practices of the 2D-LC community^28^). Here, the LC flow rate was 350 μL/min and the peak width ∼0.13 min, which yields a fraction size of 45.5 μL. Therefore, we selected commercially available samples loops of 60 μL (≈1.3 × 45.5 μL). Absolute switching times of the valves were choses to store the central part of the peak in the sample loop (dashed lines in Figure 2a).

We infused the stored fractions into the MS with a syringe pump flow rate of 150 μL/h (=2.5 μL/min), which generally gives a stable signal. This flow rate is 140 times lower than the LC flow rate, giving a theoretical 18 min of ion signal (0.13 × 140) from each fraction. To determine the exact time window with a stable signal for IRIS experiments, we performed a simple CID fragmentation experiment. During the elution of each fraction, we set the ion trap to constantly perform mass isolation (*m*/*z* 205 and *m*/*z* 195 for the tryptophan fraction and caffeine fraction, respectively) and CID fragmentation at a constant amplitude (0.3 V for tryptophan, 0.46 V for caffeine). Figure 2b shows the EICs corresponding to protonated phenylalanine and the precursor and main fragment ion of protonated tryptophan and caffeine. Here, *t* = 0 corresponds to the start of the LC run. The first 6.94 min correspond to the LC–MS analysis showing that phenylalanine (*m*/*z* 166) elutes normally as a sharp peak at the correct retention time, while at the elution times of tryptophan and caffeine (corresponding with the valve switching times) no peak is observed, because fractions are diverted into the sample loops. After filling the second sample loop (6.94 min), the valves are switched to infuse the contents of the first sample loop into the ion trap, set to mass-isolate and fragment tryptophan. For ∼40 min, signals of the precursor (*m*/*z* 205) and main fragment ions (*m*/*z* 188) of tryptophan are observed.

To determine the time window for IRIS experiments, we calculated the fragmentation yield, which should be constant at a constant CID amplitude. Figure 2c shows a stable yield between ∼18.5 and ∼36 min, so that we have ∼17.5 min to perform IRIS experiments. At 54 min, V2 was switched to infuse the contents of the second sample loop. Here, a signal for the precursor and main fragment ion of protonated caffeine was observed from ∼59–85 min (Figure 2b), and a stable yield was observed from ∼60–82 min (22 min, Figure 2c). This is longer than that for tryptophan, which may be related to the higher signal intensity for caffeine as was observed in the LC–MS analysis (Figure 2a). This is also longer than the theoretical 18 min (see above), indicating that some peak broadening takes place during the infusion of the loop contents. We note that mass isolation during loop analysis enables longer accumulations times. The MS software normalizes the ion count on the accumulation time. This does not take into consideration potential flow rate-dependent variation in ion current from the source. The magnification factor in the figure is qualitative and used for data presentation purposes.

The same valve switching times were used to repeat the experiment with IRIS. The EICs are shown in Figure 2d. Note that in this case the analysis of the loops was started somewhat later (at 10 min) than in panel b, leading to a shift of all signals by a few minutes. Here, fragmentation was induced by the IR laser (see the Method section), which was stepped through the IR fingerprint range (1900–650 cm^–1^ for tryptophan and 1900–900 cm^–1^ for caffeine, in 5 cm^–1^ steps); each point in the loop analysis indicates a different wavelength point of the laser. We performed two IR scans for protonated tryptophan and three for protonated caffeine in a single LC run. The start and the end of each IR scan are indicated in Figure 2d. In this case, fragment ions are not constantly observed but rather appear in peaks, indicating points where the IR laser is on resonance with a vibrational transition in the precursor ion. Figure 2e compares the IR spectra obtained in this manner to reference IR spectra obtained in a direct-infusion experiment, showing that the spectra match closely.

### Proof-of-Concept I: Identification of Phase I and Phase II Drug Metabolites

**Figure 3:**
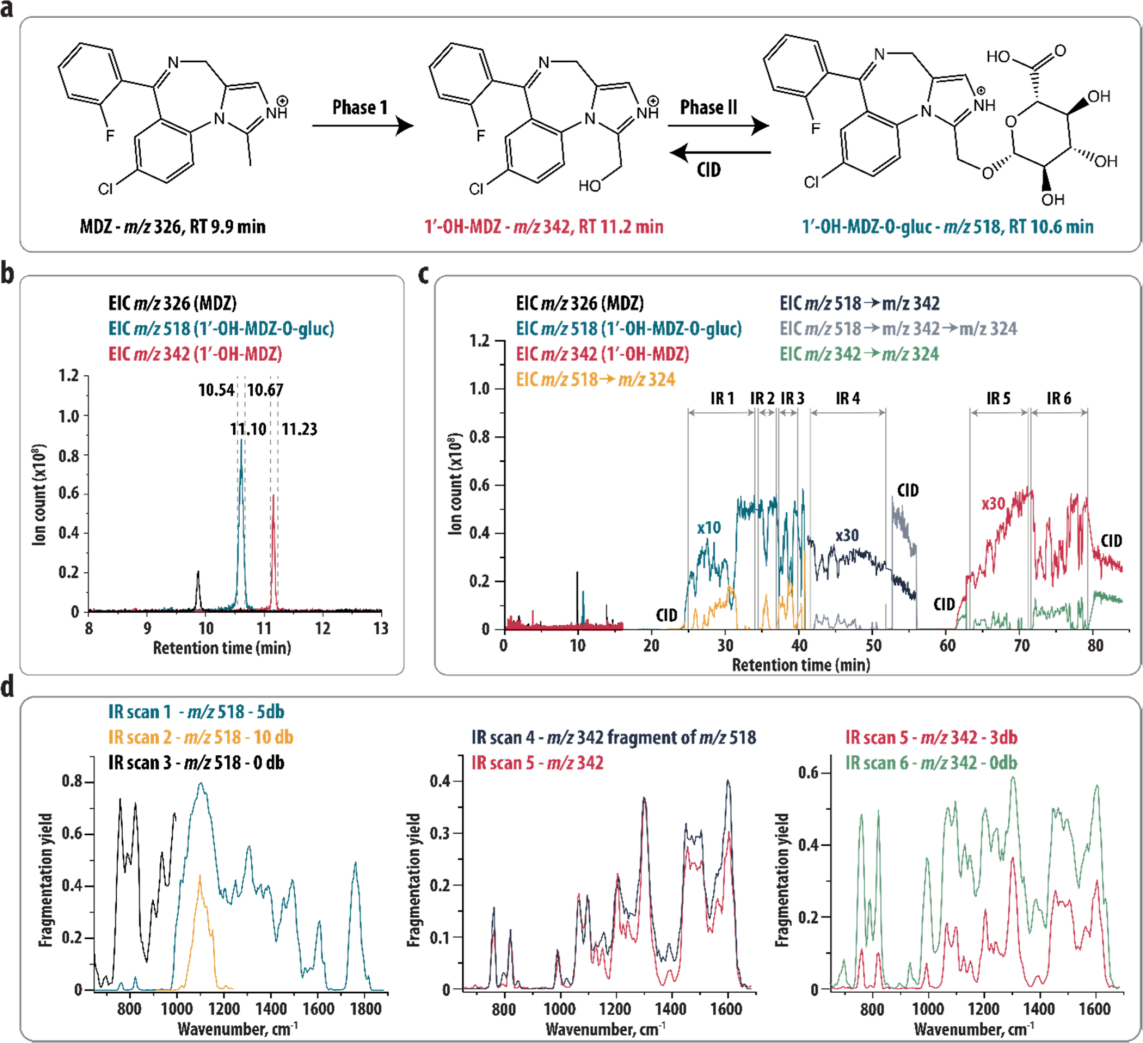
(a) Metabolism of MDZ. Phase I metabolism involves hydroxylation (+16 MW) yielding 1′-OH-MDZ, which is further metabolized in a glucuronide yielding 1′-OH-MDZ-*O*-gluc. Protonated structures are shown. (b) EICs resulting from the LC–MS analysis of an MDZ incubation sample. The elution of protonated MDZ, 1′-OH-MDZ, and 1′-OH-MDZ-*O*-gluc is observed. Dashed lines indicate the switching times of the valves leading to storage of the 1′-OH-MDZ-*O*-gluc and 1′-OH-MDZ fraction in sample loops 1 and 2, respectively. (d) EICs resulting from the analysis of the incubation sample using the heartcutting LC-IRIS set-up. The start and end of each IR scan and time windows in which CIC is performed are indicated. EICs during the analysis of the loops (>16 min) are scaled by factors of 10 and 30 for better visualization. (d) IR spectra resulting from the IRIS analysis shown in (c). IR laser intensities are labeled with the amount of laser attenuation in dB (0, 3, 5, and 10 dB correspond to 100, 50, 31.6, and 10% of the maximum laser intensity).

LC–MS is commonly applied to characterize downstream metabolites of drug compounds during drug discovery and development.^33,34^ However, using MS to identify drug metabolite structures comes with challenges, i.e., the type of metabolic transformation that takes place can be inferred from the mass difference between the drug and the metabolite, but determining the exact site of biotransformation is often difficult. We recently showed the additional value of IRIS by identifying metabolites of the drug midazolam (MDZ).^35^ MDZ undergoes a hydroxylation reaction yielding 1′-hydroxymidazolam (1′-OH-MDZ, *m*/*z* 342), which undergoes a second metabolic reaction yielding 1′-hydroxymidazolam-*O*-glucuronide (1′-OH-MDZ-*O*-gluc, *m*/z 518, see Figure 3a). Identification of the MDZ metabolites involved recording IR spectra of the two ions, but also of the glucuronide-loss CID MS/MS fragment (the aglycone) of 1′-OH-MDZ-*O*-gluc. This fragment is expected to be identical to 1′-OH-MDZ (see Figure 3a), and its IR spectrum was therefore used to establish a link between the two metabolites, revealing the position of the OH-group in 1′-OH-MDZ-*O*-gluc. Moreover, the IR absorption bands of these metabolites have a large variation in the absorption cross-section, so that multiple IR scans were recorded at different laser power settings to detect all IR features and to prevent excessive ion depletion at strong vibrational transitions. Here, we repeated the experiments on MDZ to demonstrate that all required IR spectra, involving different settings of the laser beam attenuation, can be measured from a single LC injection using the heartcutting-LC-IRIS set-up. Figure 3b contains the results of an LC-MS analysis, showing the elution of protonated MDZ (*m*/*z* 326), 1′-OH-MDZ-*O*-gluc (*m*/*z* 518), and 1′-OH-MDZ (*m*/*z* 342). The valves were set to switch at appropriate times to store the *m*/*z* 518 fraction in the first loop and the *m*/*z* 342 fraction in second loop. Figure 3c shows several EICs recorded during the heartcutting LC-IRIS experiment. Six IR scans were performed and at several time points during the analysis, CID was performed to check the signal stability (indicated in Figure 3c). During the first part of the analysis of the first loop, *m*/*z* 518 was mass-isolated and irradiated, leading to dissociation into multiple fragments. For clarity, only the EIC of the most intense fragment ion (*m*/*z* 324) is presented. Three IR scans were performed at different IR laser intensities. The resulting IR spectra (presented as the yield of all fragment ions) are shown in Figure 3d (left panel). Subsequently, *m*/*z* 518 was mass-isolated and fragmented by CID to the *m*/*z* 342 ion, which was mass-isolated and irradiated by FELIX; Figure 3c shows the EIC of the main IRMPD fragment ion at *m*/*z* 324. During the infusion of the second loop, *m*/*z* 342 was mass-isolated, and its IRIS spectrum is compared to the IR spectrum of the *m*/*z* 342 fragment ion from the *m*/*z* 518 precursor in the middle panel of Figure 3d, indicating that these ions indeed possess the same IR spectrum and hence correspond to the same structure. The IR analysis of the *m*/*z* 342 ion was repeated using a higher laser power to pick up the low-intensity bands, as shown in the right panel of Figure 3d.

### Proof-of-Concept II: Identifying Diastereomeric Biomarkers for Pyridoxine-Dependent Epilepsy

**Figure 4:**
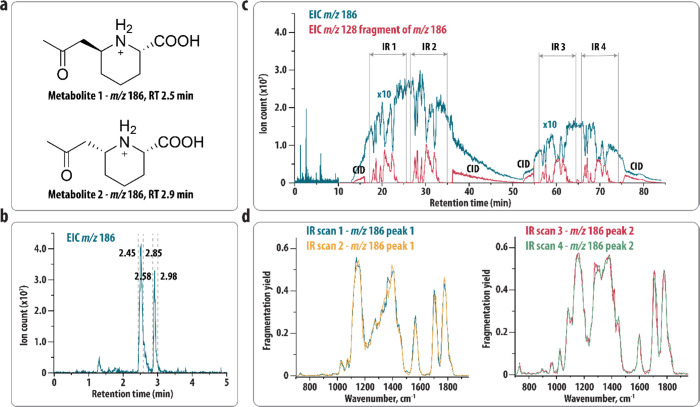
(a) Diastereomeric metabolite biomarkers (*m*/*z* 186) for PDE-ALDH7A1. (b) EICs resulting from the LC–MS analysis of a PDE plasma sample showing the elution of the two biomarkers. The dashed lines indicate the switching times of the valves leading to the storage of metabolites 1 and 2 in sample loops 1 and 2, respectively. (c) EICs resulting from the analysis of the plasma sample using the heartcutting LC-IRIS setup. The start and end of each IR scan and the time windows where CID is performed are indicated. EICs during the analysis of the loops (>10 min) are scaled by a factor of 10 for better visualization. (d) IR spectra resulting from the IRIS analysis in (c).

Untargeted metabolomics based on LC–MS is routinely employed in the search for new biomarkers for inborn errors of metabolism.^31,36^ However, identification of the detected metabolites remains a major bottleneck of this approach and the application of LC-IRIS has therefore recently been explored in this context.^22,37^ PDE-ALDH7A1 is an inborn error of metabolism leading to severe epilepsy in newborns.^38,39^ Recently, IRIS was used to identify two novel metabolites associated with PDE-ALDH7A1 (structures shown in Figure 4a).^22,37^ These biomarkers are diastereomers and have very similar retention in a reversed-phase LC separation (see Figure 4b). Obtaining isolated fractions that contain only one of the diastereomers is therefore practically challenging using an offline approach (as discussed above) and likely impossible using stop-flow chromatography.

Using the heartcutting LC-IRIS setup, the first and second metabolite peaks (both *m*/*z* 186) were stored in the first and second sample loop, respectively. Switching times of the two valves are indicated by the dashed gray lines in Figure 4b,c shows several EICs recorded during the heartcutting LC-IRIS experiment, demonstrating that we were able to obtain two IR spectra for each of the metabolites. Both metabolites primarily dissociate toward an ion with *m*/*z* 128, and the resulting IR spectra are shown in Figure 4d. These results confirm that there is no carryover between the two IR spectra; for instance, the peak at 1550 cm^–1^ in the spectrum of the first eluting feature is completely absent in the IR spectrum of the second eluting feature. In a previous study,^37^ the concentration of the two metabolites in PDE-ALDH7A1 patient plasma was determined to be ∼3 μM.

## Conclusions

In recent years, IRIS is increasingly recognized as a molecular identification tool in MS-based analytical workflows and has seen application in multiple (bio)chemical research areas. However, many analytical workflows combine MS with LC-separation, whereas the direct coupling of LC and IRIS is difficult because of the mismatching timescales of the two technologies: an IRIS run takes much longer than the width of a typical LC peak. Here, we present a heartcutting LC-IRIS setup as a novel approach to analytical LC-IRIS. Using this setup, fractions of analytes eluting from an LC column can be stored in a sample loop and infused into the MS instrument at a reduced flow rate, elongating the analyte signal to ∼20 min. This enables the acquisition of several IR spectra for each analyte, allowing for spectral averaging for improved data quality or for the acquisition of spectra under different experimental conditions (e.g., various laser pulse energies, fragment ion IR spectra, etc.). Here, we have demonstrated the approach for LC-IRIS, but it is also directly suitable for other types of MS-integrated spectroscopy experiments.^40,41^ Using additional switching valves, the setup can easily be expanded to allow the analysis of more than two analytes per LC injection.

## Supporting Information Available

IRIS spectra and extended experimental section (PDF)
