# Supplementary material for: Targeted Small-Molecule Identification Using Heartcutting Liquid Chromatography–Infrared Ion Spectroscopy
Source: Anal Chem. 2023 Feb 3;95(6):3406–13. doi: 10.1021/acs.analchem.2c04904 (PMC9933049; doi:10.1021/acs.analchem.2c04904)
Supplement: Supplementary file 1 — ac2c04904_si_001.pdf [file ac2c04904_si_001.pdf]

Supporting information

Targeted small molecule identification using heartcutting liquid chromatography–infrared ion spectroscopy

Rianne E. van Outersterp[1], Jitse Oosterhout[1], Christoph R. Gebhardt[2], Giel Berden[1], Udo F.H. Engelke[3], Ron. A. Wevers[3], Filip Cuyckens[4], Jos Oomens[1,5], Jonathan Martens[1*]

[1]Radboud University, Institute for Molecules and Materials, FELIX Laboratory, Toernooiveld 7, 6525 ED Nijmegen, The Netherlands.

[2]Bruker Daltonik GmbH & Co. KG, Fahrenheitstrasse 4, D-28359 Bremen, Germany

[3]Department of Laboratory Medicine, Translational Metabolic Laboratory, Radboud University Medical Center, 6525 GA Nijmegen, The Netherlands

[4]Drug Metabolism & Pharmacokinetics, Janssen R&D, Beerse, Belgium

[5]van't Hoff Institute for Molecular Sciences, University of Amsterdam, 1098XH Amsterdam, The Netherlands

*corresponding author

E-mail: jonathan.martens@ru.nl

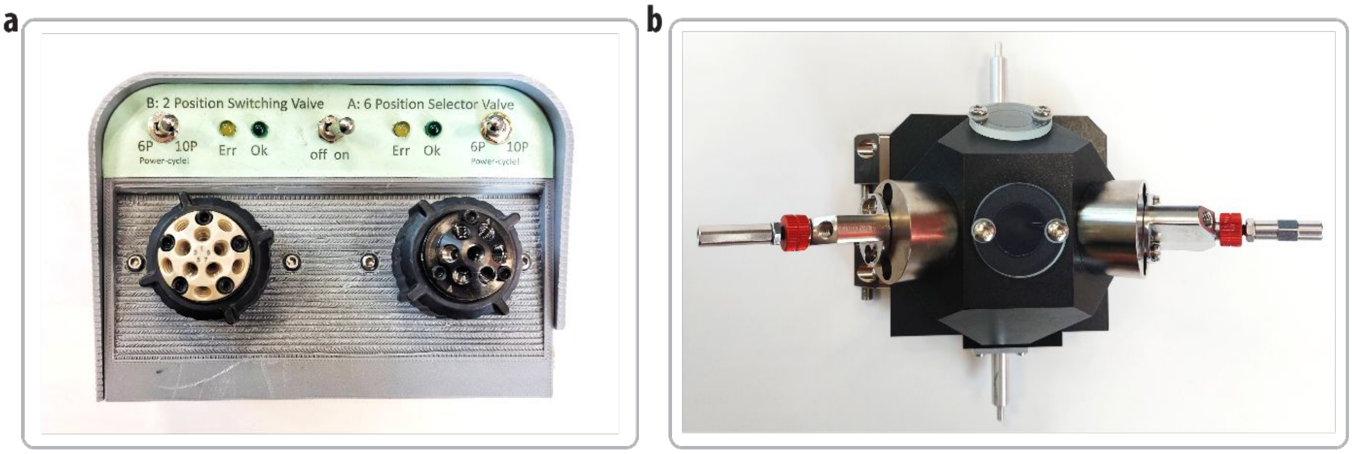

Figure S1. Photos of (a) the homebuilt valve switching box holding V1 (right) and V2 (left) and (b) modified Apollo ESI source holding two ESI sprayers.

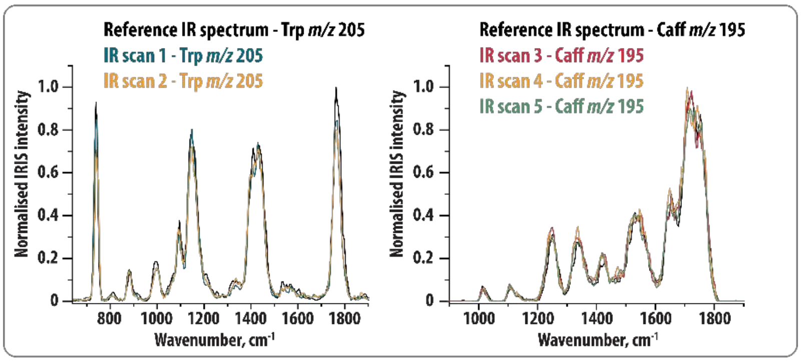

Figure S2. IR spectra resulting from the IRIS analysis shown in Figure 3d compared to reference IR spectra obtained in a direct infusion experiment. The IR intensity is computed via: IRIS intensity = ln(ΣI(precursor + fragment ions)/I(precursor ion)) and IRIS intensities are corrected for frequency-dependent laser pulse energy variations.

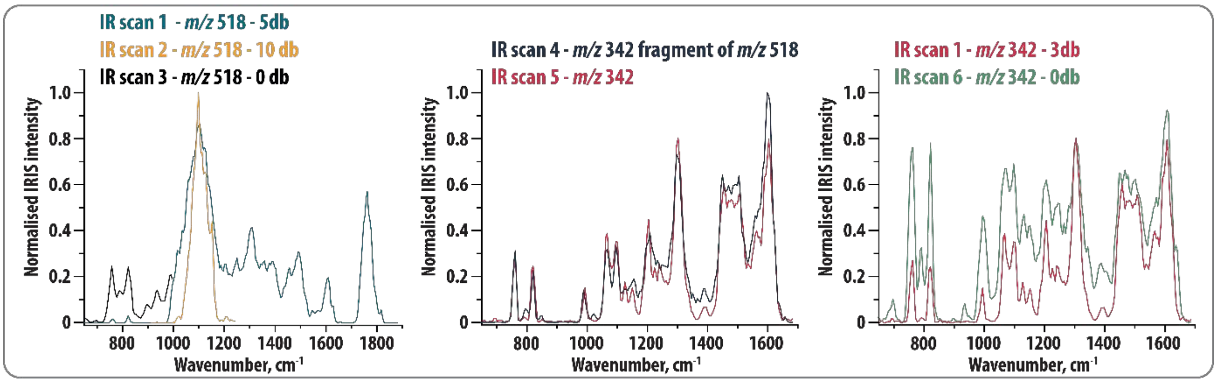

Figure S3. IR spectra resulting from the IRIS analysis shown in Figure 4c. The IR intensity is computed via: IRIS intensity = ln(ΣI(precursor + fragment ions)/I(precursor ion)) and IRIS intensities are corrected for frequency-dependent laser pulse energy variations.

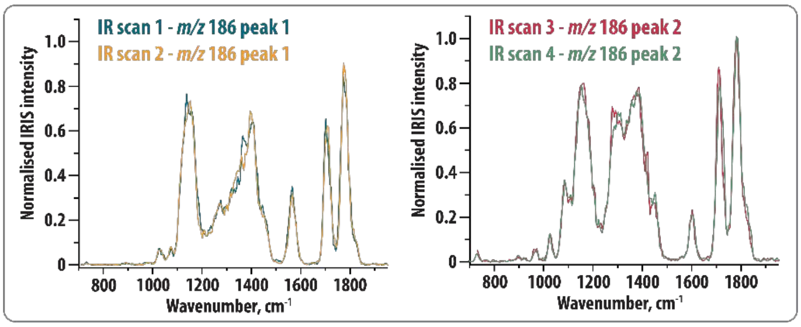

Figure S4. IR spectra resulting from the IRIS analysis shown in Figure 5c. The IR intensity is computed via: IRIS intensity = ln(ΣI(precursor + fragment ions)/I(precursor ion)) and IRIS intensities are corrected for frequency-dependent laser pulse energy variations.

Supplemental Methods

Chemicals

LC-MS grade water (H_2O) and methanol (MeOH) were obtained from Biosolve (Valkenswaard, the Netherlands). LC-MS grade acetic acid (CH_3COOH) was obtained from Fisher Scientific (Geel, Belgium). Phenylalanine (Phe), tryptophan (Trp) and caffeine (Caff) were obtained from Sigma-Aldrich (St. Louis, USA). Midazolam was purchased from Merck (Darmstadt, Germany). Acetonitrile (ACN) and formic acid (HCOOH) used for the midazolam sample preparation were obtained from Biosolve (Valkenswaard, the Netherlands). HPLC grade H_2O, MeOH, ethanol (EtOH), and HCOOH used for the plasma sample preparation procedure were obtained from Sigma-Aldrich (St. Louis, USA).

Plasma sample preparation

The plasma sample from a patient with the inborn error of metabolism pyridoxine-dependent epilepsy (PDE-ALDHA7A1), stored at -80 °C, was thawed at 4 °C. 100 µl of sample was combined with 400 µl of ice-cold MeOH/EtOH 50:50 [v/v], mixed with a vortex mixer and incubated for 20 min at 4 °C. The mixture was centrifuged for 15 min at 4 °C and 18600 g. The supernatant (350 µl) was dried in a centrifugal vacuum evaporator (Eppendorf) and reconstituted in 100 µl of H_2O/MeOH 90:10 [v/v] with 0.1% HCOOH. The sample was mixed for 15 s with a vortex mixer and centrifuged for 15 min at 18600 g (room temperature). The supernatant was used for LC-IRIS analysis.

Liquid chromatography

LC separations were performed with a Bruker Elute UHPLC system consisting of a binary pump, cooled autosampler and column oven. The outlet of the column was connected to the heartcutting LC-IRIS interface (see below). A Waters Acquity HSS T3 C18 column (100 × 2.1 mm i.d., 1.8 µm particles, 100 Å pore size) held at 40 °C and mobile phases consisting of 10 mM CH_3COOH in H2O (A) and 10 mM CH_3COOH in MeOH (B) were employed. After an initial time of 1 min at 99% A, a gradient was run to 100% B in 15 min, followed by a hold at 100% B for 2 minutes. An equilibration time of 2 minutes was used between injections. The flow rate was 0.35 ml/min and an injection volume of 2 µl was used for all samples.

ESI source modification

The ESI source installed on the ion trap receives flow from two different parts of the setup (V1-1 and V2-7, see Figure 1 in the main text). To enable this, we modified the standard Apollo ESI source of the amaZon speed ion trap MS. Standardly, the source receives flow via an ESI sprayer installed at the top of the housing and two windows are installed on the left and right sides. We removed those windows, installed ESI sprayers on both sides of the source and placed one of the windows on the top of the source (see Figure S1). This allows the ESI sprayers to be separately optimized to receive flow from two different sources at different flow rates; the needle extension of one ESI sprayer (connected to port 1 of V1) was optimized for the high flows received directly from the LC system during LC-MS analysis and the other sprayer was optimized for the lower flows received from port 7 of V2 during IRIS analysis. Additionally, the standard nebulizer gas (N2) delivered by the amaZon speed instrument was used to assist ESI at the low-flow sprayer and an additional external gas controller (E-5752-AAA, Bronkhorst HI-TEC) was installed to deliver a flow of 400 L/hr to the higher-flow sprayer. Both N_2-flows arrive in the same spray chamber and therefore they influence the ESI process at both sprayers. We determined that the best ion signals are obtained when the external gas controller (delivering N_2 to the high-flow sprayer) is turned off during the IRIS experiments (panel d and e in Figure 1 of the main text).

Collision induced dissociation and infrared ion spectroscopy experiments

All experiments were performed using a 3D ion trap mass spectrometer (Bruker, amaZon speed ETD) modified for IRIS experiments using the FELIX free electron laser (see Ref. [32]). Apart from photodissociation MS/MS experiments, we used collision-induced dissociation (CID) for a preliminary evaluation of the set-up or to monitor the signal before the start of an LC-IRIS experiment. Here, 28-40 ms of CID wasused. The CID amplitude was adapted to each ion. The CID fragmentation yield was calculated by relating the fragment and precursor ion intensities (fragmentation yield = $I_{fragment}$ / $I_{fragment}$ + $I_{precursor}$).

For IRIS experiments, FELIX was set to produce 10 Hz macropulses of duration 5-10 μs and energy 10-80 mJ (varying with wavelength) in the 650-1900 cm^{-1} region. The bandwidth was ~0.4% of the central frequency. Ions of interest were mass-isolated and irradiated using a single macropulse. When the laser is on resonance with a vibrational transition of the ions, resonant absorption occurs and leads to an increase of ion internal energy followed by unimolecular dissociation. This was detected by recording an MS spectrum after irradiation. To acquire an IR spectrum, FELIX was stepped through the wavelength range (in 5 cm^{-1} steps) while recording an MS/MS spectrum at each wavelength point (taking 6 averages at each point). Analogous to the CID experiments, IR spectra were generated by plotting the IR-induced fragmentation yield as function of laser frequency. Here, the IR wavelength was calibrated using a grating spectrometer. Usually, when comparing IRIS spectra to linear absorption spectra (such as obtained from quantum-chemical calculations), the intensity is calculated via: IRIS intensity = ln(ΣI(precursor + fragment ions)/I(precursor ion)) and IRIS intensities are corrected for frequency-dependent laser pulse energy variations.[33] IR spectra calculated in this manner can be found in the supporting information (Figure S2-S4).
